# Melanopic irradiance defines the impact of evening display light on sleep latency, melatonin and alertness

**DOI:** 10.1038/s42003-023-04598-4

**Published:** 2023-03-01

**Authors:** Isabel Schöllhorn, Oliver Stefani, Robert J. Lucas, Manuel Spitschan, Helen C. Slawik, Christian Cajochen

**Affiliations:** 1 Centre for Chronobiology, Psychiatric Hospital of the University of Basel, Basel, Switzerland; 2 Research Platform Molecular and Cognitive Neurosciences (MCN), University of Basel, Basel, Switzerland; 3 Centre for Biological Timing, Division of Neuroscience, School of Biological Sciences, Faculty of Biology Medicine and Health, University of Manchester, Manchester, UK; 4 Translational Sensory & Circadian Neuroscience, Max Planck Institute for Biological Cybernetics, Tübingen, Germany; 5 Chronobiology & Health, TUM Department of Sport and Health Sciences (TUM SG), Technical University of Munich, Munich, Germany; 6 TUM Institute for Advanced Study (TUM-IAS), Technical University of Munich, Garching, Germany; 7 Clinical Sleep Laboratory, Psychiatric Hospital of the University of Basel, Basel, Switzerland

**Keywords:** Retina, Circadian rhythms and sleep

## Abstract

Evening light-emitting visual displays may disrupt sleep, suppress melatonin and increase alertness. Here, we control melanopic irradiance independent of display luminance and colour, in 72 healthy males 4 h before habitual bedtime and expose each of them to one of four luminance levels (i.e., dim light, smartphone, tablet or computer screen illuminance) at a low and a high melanopic irradiance setting. Low melanopic light shortens the time to fall asleep, attenuates evening melatonin suppression, reduces morning melatonin, advances evening melatonin onset and decreases alertness compared to high melanopic light. In addition, we observe dose-dependent increases in sleep latency, reductions in melatonin concentration and delays in melatonin onset as a function of melanopic irradiance—not so for subjective alertness. We identify melanopic irradiance as an appropriate parameter to mitigate the unwanted effects of screen use at night. Our results may help the many people who sit in front of screens in the evening or at night to fall asleep faster, feel sleepier, and have a more stable melatonin phase by spectrally tuning the visual display light without compromising the visual appearance.

## Abstract (web summary)

An analysis of sleep patterns and melatonin concentrations in a cohort of male participants exposed to varying luminance levels commonly experienced in the evening showed that melanopic irradiance is a meaningful target for efforts to limit the impact of evening display light on the time to fall asleep, melatonin and alertness.

## Introduction

Consumption of electronic media is widespread, with many people using them for about 6–10 h a day^1,2^. In a recent study by Pham et al.^3^, 98.1% of young students reported using electronic devices within 2 h before habitual bedtime, and 62.9% used their devices in bed.

However, display light in the evening and night can cause undesirable non-image-forming (NIF) effects. It increases alertness and influences sleep architecture^4,5^. Furthermore, display light can suppress the production of the pineal hormone melatonin^4,6,7^, and may shift the endogenous circadian clock to a later time^8^. The usual light levels experienced in the evening are relatively low compared to daylight (computer screens: ~80 lx, tablets: ~40 lx, smartphones: ~20 lx, watching television: ~10 lx) yet can yield biologically potent effects. There is thus an ongoing need to define strategies to minimise unwanted NIF effects of evening visual display use.

In addition to the rods and cones, the intrinsically photosensitive retinal ganglion cells (ipRGCs) in the retina also signal information about light through the photopigment melanopsin. ipRGCs encode information about environmental illumination for diverse brain areas, primarily the internal master clock in the hypothalamus located in suprachiasmatic nuclei^9–12^. Melanopsin has a peak wavelength sensitivity of 480 nm at the retinal level^13^ before pre-receptoral filtering.

Melanopsin’s enhanced sensitivity to shorter wavelengths has led to the development of various strategies to reduce short-wavelength light exposure from displays. So-called blue-light blocking glasses help to protect from evening light-induced melatonin suppression^14^ and attenuate the activating properties of evening light^15^. Furthermore, various software-based methods are used to manipulate light settings by making them dimmer and shifting the colour balance to a more yellow set point by reducing short-wavelength content. Both filters and colour-shifting software additionally modify brightness and colour as potentially unwanted effects. It is widely assumed that the ability of these strategies to attenuate NIF responses reflects reduced melanopsin excitation. Although NIF responses to light are commonly attributed to melanopsin excitation, the selective contribution of the melanopic system to NIF effects cannot be quantified with the before-mentioned strategies, as they simultaneously change cone excitation. Thus, the magnitude of NIF effects caused by selectively modulating melanopic radiance needs further investigation. An experimental tool for selective melanopsin stimulation, without affecting colour and brightness perception, are metamers^16^.

In the present study, we exploit a five-primary display architecture to selectively modulate screen melanopic radiance while keeping cone excitation constant^17^ and produce white metameric display images that were matched in cone activation and were therefore visually indistinguishable. There are first indications that selective melanopsin modulation might affect melatonin concentration^18,19^ and alertness^20^. We set out here to use this approach to determine the relevance of melanopic irradiance in determining the NIF response amplitude across the range of lighting levels typically encountered during screen use. In addition to melatonin suppression and alertness, we aimed to assess longer-term impacts extending through to the subsequent sleep initiation process by concentrating on the time to fall asleep after lights off (i.e., sleep latency) and the melatonin concentration on the following morning.

We hypothesised that (1) evening low melanopic display light (LM) shortens sleep latency, advances the onset of melatonin secretion, and mitigates melatonin attenuation and alerting effects compared to metameric high melanopic display light (HM) and (2) NIF responses across the low light levels produced by display use in the evening intensify with increasing melanopic equivalent daylight illuminance (mEDI) levels.

## Results

**Fig. 1:**
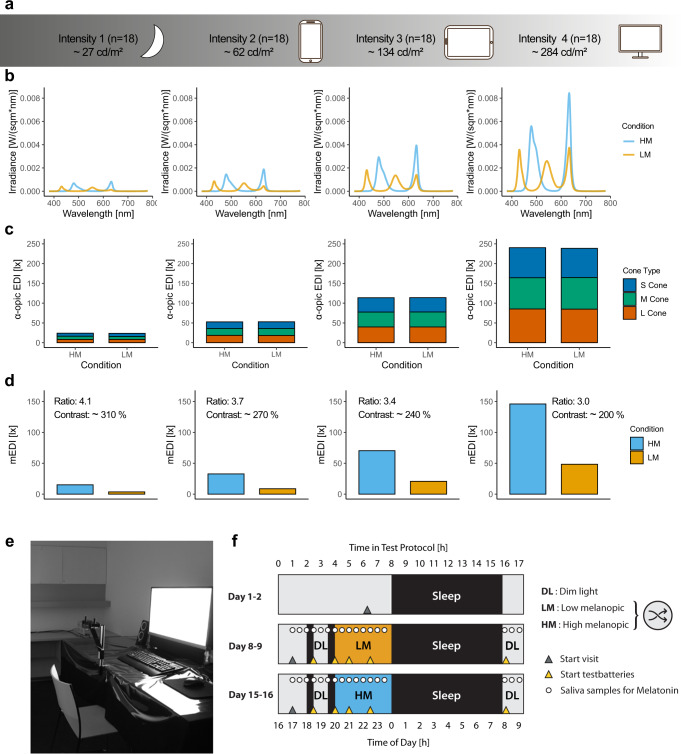
Lighting conditions and experimental protocol of the study. **a** Participants were assigned to one of the four light intensity groups (*n* = 18 per group) that differed in luminance [cd/m^2^]. **b** Irradiance of all four light intensity groups [W/(sqm*nm)]. Irradiances of the low melanopic condition (LM) are depicted with orange lines and the high melanopic condition (HM) with blue lines. **c** Summation of the alpha-opic equivalent daylight illuminance for the three cone types (CIE S 0 26) [lx] in the four intensity groups (S-cones: blue bars; M-cones: green bars; L-cones: red bars). **d** Melanopic equivalent daylight illuminance (mEDI) in the four intensity groups shows the ~200–300% melanopsin contrast (Contrast = (HM-LM)/LM [%]; Ratio = HM/LM) between the low melanopic (LM: orange bars) and high melanopic (HM: blue bars) condition. **e** Photo illustrating screen and experimental setup. Participants were sitting at a distance of 60 cm in front of the screen. **f** The study protocol consisted of one adaptation night and two 17-h blocks either under the LM or HM condition. The order of both conditions was balanced. Both experimental blocks started 7 h prior to habitual bedtime under standard fluorescent light (~67 lx). Before light exposure, there were two episodes of dark adaptation (~0.1 lx) and one dim light episode (~0–7 lx) for adaptation of photoreceptor sensitivity and to collect baseline measurements. Participants were exposed to LM or HM, respectively, for 3.5 h, starting 4 h before habitual bedtime. The light exposure was followed by an 8-h sleep episode, which was polysomnographically recorded (for details, see “Methods” section) and a 1-h dim light episode the next morning. The yellow triangles indicate the timing of the cognitive test batteries. The results of the cognitive tasks will be published elsewhere. Before, during, and in the morning after light exposure, salivary melatonin levels were measured, and subjective levels of sleepiness were rated in half-hourly intervals throughout scheduled wakefulness.

Here, we quantified melanopsin-dependent effects of evening display light on sleep latency, melatonin concentration, melatonin onset and subjective alertness (as indexed on the Karolinska Sleepiness Scale^21^). Seventy-two healthy male participants (18–35 years, mean = 24.7 ± 4.3 years) were each assigned to one of four light intensity groups (*n* = 18 per group, Fig. 1a). Supplementary Table 1 provides an overview of participants’ characteristics. Both LM and HM were matched in terms of luminance and chromaticity (S, M and L cones) (Fig. 1b, c). With the optimised display prototype, we achieved 200–300% Weber contrast for melanopsin between LM and HM (Fig. 1d). Display light exposure started 4 h before habitual bedtime (Fig. 1e, f).

### Sleep latency

**Fig. 2:**
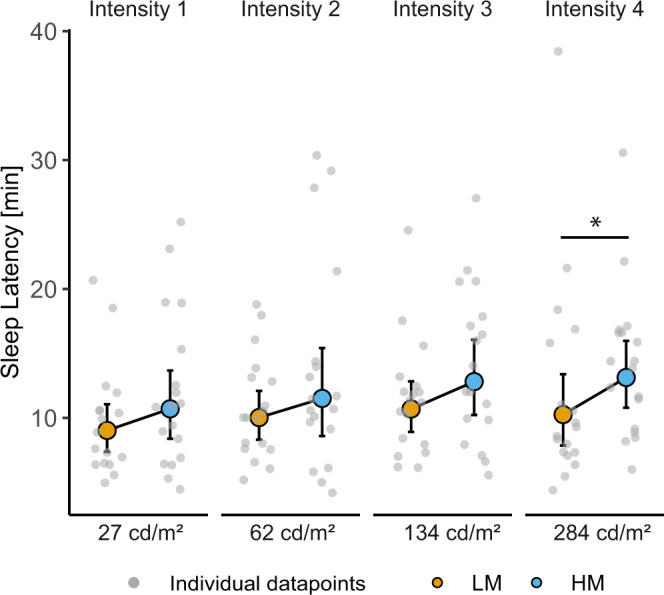
Sleep latencies to stage N2 in minutes after lights off. Depicted are back-transformed means for the low melanopic condition (LM: orange points) and the high melanopic condition (blue points) and 95% CIs (black lines) in the four light intensity groups (*n* = 18 per group, except LM intensity 1 *n* = 17). Grey points represent the individual values of participants. Black asterisks indicate the statistically significant main effect (*p* < 0.05) of the fixed factor light condition, computed in a linear mixed model.

We first examined whether sleep latency (SL) is modulated by selective melanopsin stimulation across the intensity range. Therefore, we compared LM vs HM differences in SL within the four intensity groups. In intensity group 4 (highest luminance, 284 cd/m^2^), SL was significantly reduced after LM compared to HM (*F*_1,17_ = 6.13, *p* = 0.024, large effect). In intensity group 3 (134 cd/m^2^) sleep latency tended to be shorter in LM compared to HM (*F*_1,17_ = 4.16, *p* = 0.057). The LM-HM differences in light intensity groups 1, 2 and 3 (27–134 cd/m^2^) did not show significant differences (Fig. 2, Supplementary Table 2 for all analysis of variance results and Supplementary Table 3 for 95% confidence intervals of the mean). These results indicate that selective melanopsin excitation affects sleep latency without affecting visual appearance.

### Melatonin

**Fig. 3:**
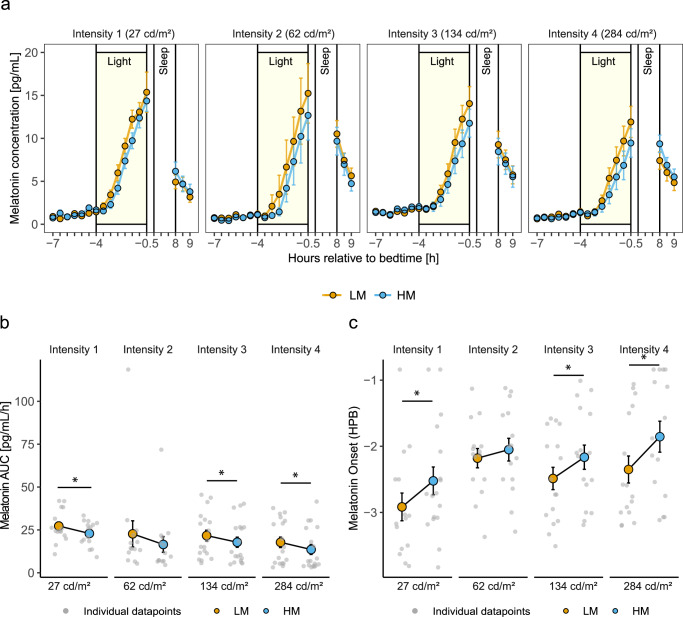
Results for melatonin during light exposure. **a** Time course of salivary melatonin concentrations in pg/mL during the low melanopic (LM: orange points and lines) and high melanopic (HM: blue points and lines) light conditions plotted against the hours relative to bedtime [h]. Depicted are means ± 1SEM of the four light intensity groups (Intensity 1: *n* = 15, intensity 2: *n* = 14, intensity 3: *n* = 18, intensity 4: *n* = 18). In total, data of seven participants were not included in the data analysis due to low melatonin concentrations during the evening (for details see “Methods” section) **b** Melatonin AUC (area under the curve) in pg/mL/h during light exposure. Depicted are the means ± 1SEM for LM (Mean: orange points; SEM: black lines) and HM (Mean: blue points; SEM: black lines). (Intensity 1: *n* = 15, intensity 2: *n* = 14, intensity 3: *n* = 18, intensity 4: *n* = 18) **c** Melatonin onset relative to bedtime (HPB: Hours prior bedtime) during LM (Mean: orange points; SEM: black lines) and HM (Mean: blue points; SEM: black lines). Depicted are means ± 1SEM (Intensity 1: LM *n* = 15, HM *n* = 17; intensity 2: LM *n* = 15, HM *n* = 15; intensity 3: LM *n* = 16, HM *n* = 16; intensity 4: LM *n* = 16, HM *n* = 15). Grey points represent the individual values of participants. The black asterisks indicate the statistically significant main effect (*p* < 0.05) of the fixed factor light condition, computed in a linear mixed model.

Endogenous levels of the pineal hormone melatonin respond to light in a dose-dependent manner^22^. We analysed whether the exposure of evening display light with ~3-fold difference in melanopsin contrast affects salivary melatonin concentration within the four intensity groups. Figure 3a shows the average time course of salivary melatonin levels per intensity group in the LM and HM conditions. Salivary melatonin levels exhibited the typical evening increase. Melatonin concentrations during light exposure were significantly higher for LM than HM in all light intensity groups (Light Condition: Intensity 1: *F*_1,210_ = 7.82, *p* < 0.01, intensity 2: *F*_1,195_ = 5.43, *p* = 0.02, intensity 3: *F*_1,254.01_ = 8.33, *p* < 0.01, intensity 4: *F*_1,254.02_ = 8.95, *p* < 0.01, small effect in all groups).

Additionally, we examined melatonin concentrations during the first hour after wake up in the morning. Melatonin concentration was significantly lower for LM than HM in light intensity groups 1 (Light Condition: *F*_1,70_ = 5.82, *p* = 0.02, medium effect) and 4 (Light Condition: *F*_1,85_ = 9.04, *p* < 0.01, medium effect), but not in light intensity groups 2 and 3.

We investigated the total melatonin production during light exposure, measuring the area under the curve (AUC). The melatonin AUC was significantly larger during LM compared to HM in intensity groups 1 (Light Condition: *F*_1,14_ = 5.49, *p* = 0.03, large effect), 3 (Light Condition: *F*_1,17_ = 4.69, *p* < 0.05, large effect) and 4 (Light Condition: *F*_1,17_ = 4.70, *p* < 0.05, large effect), but not in intensity group 2 (Fig. 3b).

Melatonin onsets were significantly earlier in LM compared to HM in light intensity groups 1 (Light Condition: *F*_1,13.12_ = 24.39, *p* < 0.001, large effect), 3 (Light Condition: *F*_1,14.24_ = 5.77, *p* = 0.03, large effect) and 4 (Light Condition: *F*_1,14.28_ = 9.31, *p* = 0.01, large effect) but not 2 (Fig. 3c). The estimated difference between LM and HM conditions was ~0.5 h in intensity groups 1, 3 and 4. Supplementary Fig. 1 shows individual melatonin onsets.

### Subjective alertness

**Fig. 4:**
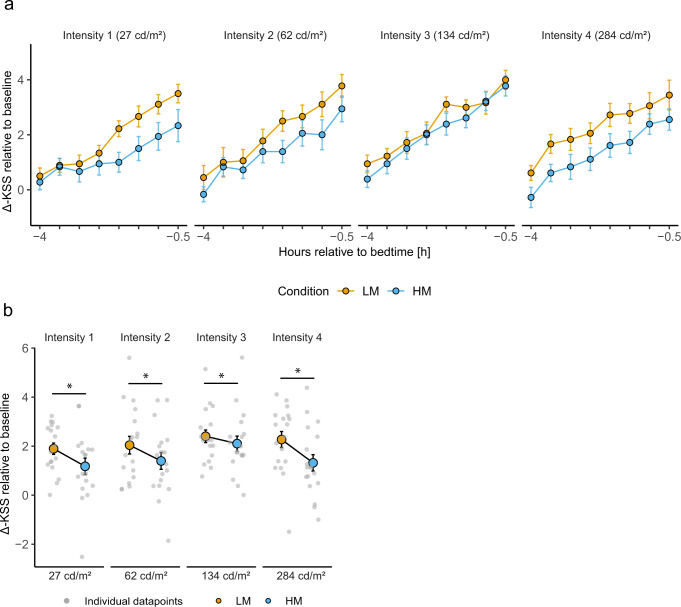
Results of subjective alerting response during light exposure. **a** Time course of subjective sleepiness ratings (KSS) during the low melanopic (LM: orange points and lines) and high melanopic (HM: blue points and lines) light conditions plotted against the hours relative to bedtime [h]. Individual sleepiness ratings were corrected with the last sleepiness rating before light exposure, showing the difference to the pre-light exposure level. Depicted are means ± 1SEM (*n* = 18 per intensity group). **b** Baseline-corrected subjective sleepiness ratings (KSS) for LM (Mean: orange points; SEM: black lines) and HM (Mean: blue points; SEM: black lines) during light exposure. Values shown are means of eight time assessments ±1SEM. Grey points represent the individual values of participants. The black asterisks indicate the statistically significant main effect (*p* < 0.05) of the fixed factor light condition, computed in a linear mixed model.

Individual sleepiness levels were normalised to the last sleepiness rating before light exposure (=Baseline) and are expressed as a change score (For uncorrected values, see Supplementary Fig. 2 and for analysis of variance results of subjective sleepiness ratings before light exposure and in the morning, see Supplementary Table 4). Subjective sleepiness levels exhibited a typical evening profile with increased sleepiness in the late evening (Fig. 4a). Baseline-corrected sleepiness levels during light exposure were significantly higher in LM than HM in all light intensity groups (Light Condition: Intensity 1: *F*_1,254.03_ = 21.69, *p* < 0.001, medium effect, intensity 2: *F*_1,255_ = 14.68, *p* < 0.001, small effect, intensity 3: *F*_1,255_ = 4.33, *p* = 0.04, small effect, intensity 4: *F*_1,255_ = 38.60, *p* < 0.001, medium effect) (Fig. 4b). There was no significant interaction between Light Condition and Session.

### mEDI as a predictor for sleep latency, melatonin AUC, melatonin onset and subjective alertness

**Fig. 5:**
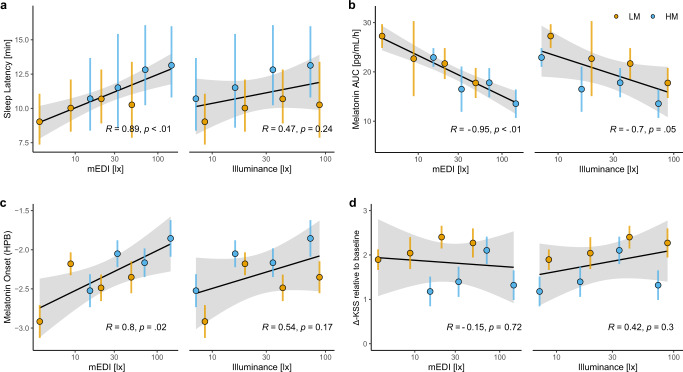
Dose-response relationships with log10-transformed mEDI [lx] and photopic illuminance [lx]. Averages per light condition over all light intensity groups entered the calculation of the regression models. Dose dependencies were calculated for **a** Sleep latency [min], depicted are back-transformed means and 95% CIs (*n* = 18 per group, except LM intensity 1 *n* = 17) and the regression line (Mean values of all light conditions, *n* = 8), **b** Melatonin area under the curve (AUC) [pg/mL/h], depicted are means ± 1SEM groups (Intensity 1: *n* = 15, intensity 2: *n* = 14, intensity 3: *n* = 18, intensity 4: *n* = 18) and the regression line (Mean values of all light conditions, *n* = 8), **c** Melatonin onset in hours prior individual bedtime (HPB), depicted are means ± 1SEM (Intensity 1: LM *n* = 15, HM *n* = 17; intensity 2: LM *n* = 15, HM *n* = 15; intensity 3: LM *n* = 16, HM *n* = 16; intensity 4: LM *n* = 16, HM *n* = 15) and the regression line (Mean values of all light conditions, *n* = 8), **d** Baseline-corrected subjective sleepiness (KSS), depicted are means ± 1SEM (*n* = 18 per intensity group) and the regression line (Mean values of all light conditions, *n* = 8). Orange points show means of the low melanopic and orange lines 95% CIs or SEM. Blue points depict means of the high melanopic and blue lines 95% CIs or SEM. The grey bands represent the 95% confidence interval limits.

Lighting standards and guidelines as well as study results on NIF effects of light have mainly been based on photopic light levels. However, recently, there has been mounting evidence that melanopic light levels (mEDI) are a better metric for predicting melatonin suppression by light than photopic lux^18,19^. We used linear regression analyses to investigate if mEDI levels and/or photopic illuminance could predict sleep latency, melatonin AUC, melatonin onset and alertness. For the analysis, we used the mean values of the measured variables calculated for the different light stimulations. We found a significant correlation between log_10_ mEDI and SL, such that higher mEDI levels were associated with a prolonged time to fall asleep (Adj. *R*^2^ = 0.77, *p* = 0.001) (Fig. 5a). There was about a 50% increase in mean sleep latency between the minimal LM condition in intensity 1 (~5 lx mEDI) and the maximal HM condition in intensity 4 (~150 lx mEDI).

Furthermore, the regression was statistically significant for melatonin AUCs (Adj. *R*^2^ = 0.89, *p* < 0.001), such that mean AUCs decreased with higher mEDI. Between the extreme light settings LM 1 (~5 lx mEDI) and HM 4 (~150 lx mEDI) there was about a 50% reduction in mean melatonin AUC (Fig. 5b).

Grouping the data for melatonin onset according to the mEDI resulted in a significant dose-response relationship, with increasing mEDI, the mean melatonin onset was shifted to a later time (Adj. *R*^2^ = 0.57, *p* = 0.02) (Fig. 5c). There was a delay of about one hour between the extreme light settings ~5 lx mEDI (LM 1) and 150 lx mEDI (HM 4).

There was no significant regression between illuminance and either melatonin AUC, sleep latency or melatonin onset, confirming that illuminance is a poor predictor of NIF response amplitude in this study.

Perhaps surprisingly given the differences that we had observed between LM and HM for subjective alertness (Fig. 4), we did not detect a significant dose-response relationship for subjective alertness when plotted against either mEDI or illuminance (Fig. 5d). This implies that while modulating melanopic irradiance can alter subjective alertness, it does not do so in an intensity-dependent manner over the range of conditions tested here.

## Discussion

First, we investigated whether evening display light has a selective melanopsin-dependent impact on sleep latency, melatonin concentration and subjective alertness levels in the evening. Second, we aimed to observe whether mEDI is a good predictor for the observed variables. Compared to high levels of melanopsin excitation, we found that low melanopsin excitation leads to shorter sleep latency, an earlier melatonin onset, less melatonin suppression, and lower alertness levels manifested already at a rather low light intensity in the evening (<10 lx).

To the best of our knowledge, we provide the first evidence that selective modulations in melanopsin activity alter sleep latency. Exposure to a low melanopic display light was associated with a shorter duration to fall asleep than after exposure to a high melanopic display light. This result indicates that melanopsin significantly contributes to humans’ sleep onset process, corroborating findings in melanopsin knockout mice^23–25^. Furthermore, we found a significant dose-response relationship between mEDI and sleep latency across a relatively low illuminance range (~ 5–150 lx mEDI). To the extent of our knowledge, such a relationship has not been previously described. Still, it corroborates findings from a meta-analysis on dose-dependent effects of prior light exposure on polysomnographically assessed sleep^26^.

Compared to high, low melanopic display light resulted in higher melatonin concentrations (i.e., less melatonin suppression by light) in all light intensity groups. Our data support the results of Allen et al.^20^ and Souman et al.^18^, who showed that melatonin concentration could be selectively modulated depending on the amount of melanopsin excitation under metameric light conditions. Interestingly, we observed significant LM vs. HM differences in melatonin concentration already under dim light conditions. However, the effect sizes were relatively small, probably due to the large interindividual differences in melatonin concentrations.

Morning melatonin concentrations were significantly higher in LM compared to HM in the lowest (Intensity 1) and highest light intensity group (Intensity 4). Furthermore, melatonin onset was significantly earlier during LM than HM in three light intensity groups (1, 3 and 4). This reveals that evening light exposure across this range (~10–90 lx) can have long-lasting impacts on human melatonin levels, extending at least to the following morning. While we cannot address the mechanisms of such an effect in our study, one plausible possibility is that it reflects a change in the circadian phase, with lower evening mEDI producing an earlier circadian phase as expected for a less active engagement of the delaying portion of the phase response curve. Previous research has modelled several dose-response curves for melatonin suppression^19,27^. Our findings indicate a dose-dependent decrease in melatonin concentration (AUC), with increasing mEDI levels corroborating earlier findings. Moreover, we found significant dose-dependent relationships between melatonin onset and mEDI. To the best of our knowledge, this is the first study, showing a dose-dependent relationship between display light and melatonin onset.

Metameric light with low melanopsin excitation induced a smaller alerting response than high melanopic light across all four intensities. Interestingly, we could not find a significant dose-dependent relationship between mEDI and subjective alertness. Since the dose-response curve of Cajochen et al.^28^ showed an increase in subjective alertness only starting above illuminance levels of about 100 lx, the illuminance of our study might have been too low to see dose-dependent effects of mEDI on subjective alertness. However, in a meta-analysis, Hommes et al.^29^ showed a better fit for melanopic than photopic illuminance and an increase of subjective alertness already at ~20 lx mEDI when comparing data sets of several studies to dim light. Although the last subjective sleepiness rating before light exposure did not yield significant differences among the four intensity groups (Supplementary Table 4), interindividual differences in subjective sleepiness before light exposure may be a possible reason why we could not find a dose dependency. Alertness might be a less robust and more complex measure for the non-visual effects of light compared to other physiological measures (e.g., melatonin) since it is not only regulated by the circadian timing system but also the homoeostatic sleep drive and potentially is subject to cognitive influences^30,31^.

Recently, 10 lx mEDI was recommended as the maximum light level in the pre-sleep environment starting 3 h before habitual bedtime^32^. Our finding of significant differences in melatonin and alertness between LM and HM at the dimmest setting (4 vs 15 lx mEDI) confirms that modulations around the recommended 10 lx mEDI threshold level can make a difference to NIF response amplitude. We are aware that the light conditions might not have been metameric for each participant, as the spectra were matched to the 10° Standard Observer using CIE S026 (Commission International de l’Eclairage (CIE))^33^ and not individually adjusted. However, using the same light conditions allowed us to keep the nominal luminance, colour and melanopsin contrast constant across the participants in the four light intensity groups (for approximation of cone intrusion, see Supplementary Note 1: Cone Intrusion and Supplementary Fig. 3).

Another limitation is the small number of participants. This could be one reason why effects on melatonin AUC and onset could not be found for all intensity groups. There were large interindividual differences in melatonin concentration and especially in light intensity group 2, several subjects (*n* = 4) did not show an evening melatonin increase above 5 pg/mL (Supplementary Fig. 4).

It has been shown that exposure to bright light during the day can mitigate non-visual effects on melatonin in the evening^34–36^. Therefore, we implemented a dim light and two dark adaptation periods before the screen light exposure. However, differences in light exposure (>7 h before habitual bedtime, before the participants entered controlled light conditions in our laboratory) and season were not controlled and may have contributed to variance in our dataset. Information about time spent outdoors on experimental days and seasonal distribution of the study appointments are provided in Supplementary Table 5 and Supplementary Fig. 5.

Besides ipRGCs and cones also rods contribute to NIF effects^37^. However, silencing rods allows a melanopsin contrast of less than 1/3 of those achieved here due to overlapping spectral sensitivities of rods and melanopsin^17^. Thus, this circumstance makes it almost impossible to distinguish between melanopsin and rhodopsin activation. On this account, our light settings were not matched for rods independently. We assume that in the highest three intensity groups (groups 2, 3 and 4), the results were mainly melanopsin-driven since the rod saturation threshold according to Adelson et al.^38^ was reached (see calculation scotopic retinal illuminances, Supplementary Table 6)^39^. Nevertheless, given the very similar spectral sensitivity of rods and melanopsin it is unsurprising that we found dose-response relationships also for rhodopic EDI (Supplementary Fig. 6). For all outcome measures, associations for rhodopic EDI were lower than for mEDI, confirming that mEDI is at least as good a predictor of response amplitude. Moreover, these two qualities would strongly covary in any realistic lighting scenario making the distinction of minimal practical^20^.

We did not include female participants to avoid the potential effects of the menstrual cycle on sleep^40^ and melatonin secretion^41^. Additionally, we excluded people older than 35 years in order to reduce variance in the effective retinal stimulus, which results from differences in lens transmittance^42–44^. Therefore, our results cannot be generalised to women, teenagers and older age groups. Children and adolescents show high light sensitivity and increased melatonin suppression even at low light levels^45,46^. According to Higuchi et al.^47^, the percentage of melatonin suppression by light was almost twice as high in children compared to adults. Thus, the potential role of metameric light to protect children and adolescents from unwanted NIF effects in the evening warrants investigation. Future work is needed to validate our results also in women, including older age groups and in the field.

The NIF impact of visual displays is defined by mEDI even across the rather low irradiances typically produced by screen use. As a result, low melanopic display light reduces sleep latency, facilitates the rise of the pineal hormone melatonin, allows earlier melatonin onset in the evening and lower melatonin concentrations in the morning, and decreases alertness in the evening. We think our results have societal relevance as many of us spend hours in front of screens in the evening or even at night. Thus, reducing mEDI represents a viable strategy for mitigating unwanted effects of evening screen use, holding the promise that achieving this goal may be possible while minimising concomitant impacts on visual appearance.

## Methods

### Ethical approval

The study was carried out in the Centre for Chronobiology in Basel (Switzerland), between December 2019 and July 2021, interrupted between mid-March and mid-May 2020 due to the global COVID-19 pandemic. The experimental protocol, screening questionnaires and consent form were approved by the Ethics Committee northwest/central Switzerland (2019-00571) and conformed to the Declaration of Helsinki. All participants provided written informed consent.

### Study protocol

The in-laboratory protocol of the study consisted of two separated 17-h blocks, which participants spent in constant environmental conditions (i.e., room temperature ~ 21 °C) with no external time cues, including clocks, smartphones and daylight. The washout period between the two in-laboratory sessions was at least 1 week to avoid carry-over effects of the prior light condition. Potential masking effects of meals were avoided by providing the same content of food and similar amounts of calories at the same time to each participant. Furthermore, participants reduced their activities and movements by constantly sitting on a chair during scheduled wakefulness and additionally being in a chinrest during the cognitive tasks. Participants were instructed to stand up for scheduled toilet breaks in ca. 1.5-hour intervals. The study protocol started 7 h prior habitual bedtime under ~67 lx of fluorescent light (Philips Master TL5 HO 54 W/830, CRI 80, 3000 K) for about one hour, followed by two adaptation episodes of complete darkness (no room light, black screen background for 25 and 20 min), separated by one dim light episode (~0–7 lx during cognitive tasks). Participants wore orange-tinted glasses during dinner time and toilet breaks. During task-free intervals participants listened to prepared audiobooks and were instructed to look at the centre of the monitor with continuously open eyes (i.e., they were asked to reduce eye blinks).

### Participants

Healthy males between 19 and 35 years were included in the study. Exclusion criteria comprised body mass index (BMI) <19 or >26, medication, drug and nicotine consumption, shift work <3 months and transmeridian travel (>2 time zones) <1 month prior study start, mother tongue other than German. Because of the use of an eye-tracking device spectacle wearers could not be included. Since auditory tasks were used, volunteers with hearing loss and tinnitus were excluded. After a telephone screening, 112 participants completed the General Medical Questionnaire, the Horne–Östberg Morningness-Eveningness Questionnaire (MEQ)^48^, the Pittsburgh Sleep Questionnaire (PSQI)^49^, the Epworth Sleepiness Scale (ESS)^50^, the Beck Depression Inventory (BDI-II)^51^, Munich Chronotype Questionnaire (MCTQ)^52^ by use of the online-tool REDCap^53,54^. People with extreme Chronotypes (MEQ score: ≤30 and ≥70) and poor sleep quality (PSQI score > 5) were excluded from the study. Ninety participants were invited for a habituation night, which included a physical examination by a physician in charge. Participants who showed poor sleep efficiency (SE < 70%) and sleep disorders like sleep apnoea (apnoea index > 10), as well as periodic limb movements (PLMS > 15) during the habituation night were excluded from further participation in the study. Additionally, participants underwent an ophthalmic screening by a graduated optometrist to exclude volunteers with visual impairments. Visual acuity was tested with the Freiburg Visual Acuity Test (FrACT) Version 3.10.2^55^, colour vision with Ishihara^56^ as well as the 100-hue^57^ and stereo vision by use of Lang II^58^. We excluded five participants with a monocular visual acuity <0.5, with colour vision deficiencies (Ishihara < 17 of 21 plates, 100-hue error score > 40) or with reduced stereoscopic vision (Lang II < 200 arc seconds).

Starting 1 week prior to the study, volunteers were requested to abstain from alcohol and caffeine consumption. Furthermore, they were instructed to keep a regular sleep-wake rhythm (self-selected bedtimes ± 30 min, ~8 h sleep per night). Compliance with this outpatient segment of the study was verified using wrist-actigraphs (ActiGraph LL.C, Pensacola, FL, USA) and self-reported sleep logs deployed on a web survey platform hosted at the University of Basel (REDcap)^53,54^. A comprehensive toxicological screening of urine for drug abuse was carried out before both study conditions. In total, 72 participants completed the two study conditions.

### Display setup

Since it is not possible to generate metameric display light with a standard LED screen with three primaries, Dr. Achim Pross developed a multiprimary display^59^. We spectrally optimised his solution and integrated five LED types in the backlight of a monitor (27-inch Full-HD IPS HP-Display) for our study. Thus, we were able to produce different 2*3 primary images. In the HM setting, we used LEDs with 480, 500, and 630 nm, while in the LM setting we used LEDs with 430, 550, and 630 nm as the dominant wavelengths. The metameric screen was designed and calibrated using the method of silent substitution^17^ implemented in Matlab (The Mathworks, Natick, MA). The light settings were matched in terms of cone excitation (L, M, S) based on the 10^°^ cone fundamentals using CIE S026 (Commission International de l’Eclairage (CIE))^33^. Adjusting these five LEDs allowed for a ~200–300% melanopsin contrast between the LM and HM. Contrasts were larger in lower irradiances due to spectral shifts of the LEDs at different intensity levels. Calibration measures were performed with JETI spectroval 1501 (JETI Technische Instrumente GmbH, Jena, Germany) at eye level at 60 cm distance from the screen. The spectrometer was inclined to the centre of the monitor at an angle of 15° (for an overview of the light measurements see Supplementary Table 7). Irradiance measures were used for calculating mEDI values. For the relevant irradiance and radiance measures, see Supplementary Data 1.

### Polysomnography

Sleep electroencephalographic activity was recorded continuously with the Vitaport Ambulatory system (Vitaport-3 digital recorder TEMEC Instruments BV, Kerkrade, the Netherlands). Twelve electroencephalographic derivations referenced against linked mastoids, two electrooculograms, one submental electromyogram, and one electrocardiogram were recorded. All signals were low pass filtered at 30 Hz at a time constant of 1 Hz. Sleep latency to sleep stage 2 (SL 2) was visually scored per 30-s epochs by two independent scorers according to standard criteria of the American Academy of Sleep Medicine^60^. Sleep latency was the primary endpoint of our study. Further polysomnographic analyses will be published elsewhere.

### Salivary melatonin

Salivary samples were collected every 30 min in the evening until 30 min before bedtime as well as in the morning, starting 5 min after scheduled wake-up. A direct double antibody radioimmunoassay was used for the determination of melatonin (RK-DSM2^61^). The minimum detectable dose of melatonin (analytical sensitivity) was determined to be 0.2 pg/mL, while the functional least detectable dose (limit of quantitation) in the saliva is 0.9 pg/mL. The measurements were performed by a third-party service laboratory (NovoLytiX GmbH, Witterswil, Switzerland) according to the Instructions of Use of the RK-DSM2, version 2020-12-22. The AUC of melatonin was calculated during the light exposure using the trapezoid method. Melatonin onset was used as a phase marker of the circadian clock and was calculated using the objective hockey-stick method^62^.

### Subjective alertness

The Karolinska Sleepiness Scale (KSS) was used for the assessment of the subjective alerting response^21^. For statistical analysis of alertness levels during light exposure, ratings during light exposure were corrected with the last alertness rating before light exposure.

### Statistics and reproducibility

Seventy-two healthy male participants were each assigned to one of four light intensity groups (*n* = 18 per group). In the sleep latency analyses, we included 143 nights (*n* = 18 per group, except LM intensity 1 *n* = 17). Due to technical issues, we lost the sleep electroencephalographic recording of one night. Seven participants in the light intensity groups 1, 2 and 3 were excluded from further melatonin concentration analyses, because the maximal evening melatonin levels were below 5 pg/mL. We did not exclude participants of group 4 with melatonin concentrations below this threshold, because according to the dose-response curve of Zeitzer et al., we expected a relevant melatonin suppression^22^. In total, the melatonin data of three participants in intensity group 1 and four participants in group 2 were excluded from the melatonin concentration analysis (for melatonin analyses of the full dataset see Supplementary Table 8, Supplementary Fig. 7 and Supplementary Fig. 8). Participants with missing samples were nevertheless included in the analysis (Total number of participants included in the analyses: Intensity 1: *n* = 15, intensity 2: *n* = 14, intensity 3: *n* = 18, intensity 4: *n* = 18). We could only detect melatonin onsets in 125 melatonin profiles during light exposure (Intensity 1: LM *n* = 15, HM *n* = 17, intensity 2: LM *n* = 15, HM *n* = 15, intensity 3: LM *n* = 16, HM *n* = 16; intensity 4: LM *n* = 16, HM *n* = 15). In 19 evening melatonin profiles, we could not detect any melatonin onset during light exposure. Either because the melatonin onset already occurred before the light exposure (*n* = 9) or the hockey-stick method could not detect a clear time point for the melatonin onset (*n* = 10).

All statistical analyses were conducted in R (Version 4.1.1, R Core Team, 2021). The primary aim of the present study was to investigate the influence of melanopsin contrast on sleep latency (i.e., primary endpoint). In addition, the influence of melanopsin contrast on melatonin concentration and subjective levels of sleepiness was investigated. The minimum irradiance at which the melanopsin contrast impacts sleep latency, melatonin concentration and subjective levels of sleepiness in a metameric light setting is not known yet. Therefore, linear mixed model analyses (LMMs) were performed separately for each light intensity group. For all variables, Light Condition was included as a fixed effect and repeated measures per participant were modelled as a random intercept. LMMs were followed by an ANOVA (Type III) function. We used log-transformed sleep latency values for all our analyses. However, we retransformed mean log-sleep latency values for visualisations in the figures.

The design of the LMM for melatonin concentrations and subjective alertness levels comprised the factor Time (17 levels for Melatonin and 16 levels for KSS-Ratings). LMMs were calculated using the packages lme4 and lmerTest. As an effect size measure, partial omega squared (ωp^2^) was calculated using the effectsize package. It can be interpreted as follows: small effect: ω_p_^2^ ≥ 0.01, medium effect: ω_p_^2^ ≥ 0.06, large effect: ω_p_^2^ ≥ 0.14. A *p*-value < 0.05 was considered to indicate statistical significance.

Linear regression models were used to describe the relationship between mEDI and the observed variables. Group means were used to calculate these models over all light settings.

### Reporting summary

Further information on research design is available in the Nature Portfolio Reporting Summary linked to this article.

## Supplementary information

Supplementary Information

Description of Additional Supplementary Files

Supplementary Data

Reporting Summary

## Data Availability

The source data behind the graphs in the paper is provided as Supplementary Data 1.
