# Supplementary material for: Melanopic irradiance defines the impact of evening display light on sleep latency, melatonin and alertness
Source: Commun Biol. 2023 Mar 1;6:228. doi: 10.1038/s42003-023-04598-4 (PMC9974389; doi:10.1038/s42003-023-04598-4)
Supplement: Supplementary file 1 — Supplementary Information [file 42003_2023_4598_MOESM1_ESM.pdf]

Supplementary Information

Melanopic irradiance defines the impact of evening display light on sleep latency, melatonin and alertness

Isabel Schöllhorn[1,2], Oliver Stefani[1,2], Robert J. Lucas[3], Manuel Spitschan[4, 5, 6], Helen C. Slawik[7], Christian Cajochen[1,2]

1 Centre for Chronobiology, Psychiatric Hospital of the University of Basel, Basel, Switzerland

2 Research Platform Molecular and Cognitive Neurosciences (MCN), University of Basel, Basel, Switzerland

3 Centre for Biological Timing, Division of Neuroscience, School of Biological Sciences, Faculty of Biology Medicine and Health, University of Manchester, Manchester, UK

4 Translational Sensory & Circadian Neuroscience, Max Planck Institute for Biological Cybernetics, Tübingen, Germany

5 Chronobiology & Health, TUM Department of Sport and Health Sciences (TUM SG), Technical University of Munich, Munich, Germany

6 TUM Institute for Advanced Study (TUM-IAS), Technical University of Munich, Garching, Germany

7 Clinical Sleep Laboratory, Psychiatric Hospital of the University of Basel, Basel, Switzerland

Corresponding author: Prof. Dr. Christian Cajochen, christian.cajochen@upk.ch

Descriptives	Intensity	N	mean	sd
Age	1	18	24.50	3.81
	2	18	25.39	5.45
	3	18	24.33	4.26
	4	18	24.67	3.51
PSQI	1	18	2.5	1.43
	2	18	2.72	1.13
	3	18	3.06	1.30
	4	18	3.17	0.99
MCTQ MS-Fc	1	18	4.41	0.50
	2	18	4.43	1.06
	3	18	4.07	0.73
	4	18	4.38	0.87
MEQ	1	18	56.33	9.87
	2	18	54.33	8.87
	3	18	55.50	8.28
	4	18	55.22	8.59
ESS	1	18	5.28	3.37
	2	18	5.33	2.81
	3	18	5.11	2.19
	4	18	5.94	3.69
BDI-II	1	18	3.11	3.34
	2	18	3.00	3.05
	3	18	3.17	4.99
	4	18	4.06	5.12
Visual Acuity Monocular left	1	17	1.11	0.40
	2	17	1.39	0.37
	3	18	1.10	0.37
	4	18	1.14	0.30
Visual Acuity Monocular right	1	17	1.14	0.43
	2	17	1.41	0.42
	3	18	1.17	0.53
	4	18	1.22	0.38
Visual Acuity Binocular	1	17	1.56	0.40
	2	17	1.71	0.32
	3	18	1.54	0.39
	4	18	1.53	0.44
100-Hue	1	18	14.22	10.10
	2	18	18.00	12.37
	3	18	16.11	11.20
	4	18	17.56	10.90

Supplementary Table 1: Participants' characteristics for the four light intensity groups. Data are expressed as mean ± SD. PSQI: Pittsburgh Sleep Questionnaire; MCTQ: Munich Chronotype Questionnaire; MS-Fc: Corrected midpoint on free days; MEQ: Horne-Östberg Morningness-Eveningness Questionnaire; ESS: Epworth Sleepiness Scale; BDI-II: Beck Depression Inventory

**Sleep latency**	Light condition (LM vs HM)	Time	Light condition * Time
Intensity 1 (27 cd/m^2)	$F_{1,16.122}$ = 2.02, p = 0.17		
Intensity 2 (62 cd/m^2)	$F_{1,17}$ = 1.20, p = 0.29		
Intensity 3 (134 cd/m^2)	$F_{1,17}$ = 4.16, p = 0.057		
Intensity 4 (284 cd/m^2)	$F_{1,17}$ = 6.13, **p = .02**, ω_p^2=0.21		
**Melatonin concentration during light exposure**			
Intensity 1 (27 cd/m^2)	$F_{1,210}$ = 7.82, **p < .01**, ω_p^2=0.03	$F_{7,210}$ = 79.76, **p < .001**, ω_p^2=0.72	$F_{7,210}$ = 0.56, p = 0.79
Intensity 2 (62 cd/m^2)	$F_{1,195}$ = 5.43, **p = .02**, ω_p^2=0.02	$F_{7,195}$ = 23.04, **p < .001**, ω_p^2=0.43	$F_{7,195}$ = 0.27, p = 0.97
Intensity 3 (134 cd/m^2)	$F_{1,254.01}$ = 8.33, **p < .01**, ω_p^2=0.03	$F_{7,254.01}$ = 61.82, **p < .001**, ω_p^2=0.62	$F_{7,254.01}$ = 1.12, p = 0.35
Intensity 4 (284 cd/m^2)	$F_{1,254.02}$ = 8.95, **p < .01**, ω_p^2= 0.03	$F_{7,254.02}$ = 38.58, **p < .001**, ω_p^2= 0.5	$F_{7,254.02}$ = 1.10, p = 0.36
**Melatonin concentration in the morning**			
Intensity 1 (27 cd/m^2)	$F_{1,70}$ = 5.82, **p = .02**, ω_p^2=0.06	$F_{2,70}$ = 19.20, **p < .001**, ω_p^2=0.33	$F_{2,70}$ = 1.61, p = 0.21
Intensity 2 (62 cd/m^2)	$F_{1,65}$ = 1.17, p = 0.28	$F_{2,65}$ = 17.59, **p < .001**, ω_p^2=0.33	$F_{2,65}$ = 0.04, p = 0.96
Intensity 3 (134 cd/m^2)	$F_{1,85}$ = 1.17, p = 0.28	$F_{2,85}$ = 15.29, **p < .001**, ω_p^2=0.25	$F_{2,85}$ = 0.12, p = 0.89
Intensity 4 (284 cd/m^2)	$F_{1,85}$ = 9.04, **p < .01**, ω_p^2=0.08	$F_{2,85}$ = 23.42, **p < .001**, ω_p^2=0.34	$F_{2,85}$ = 1.06, p = 0.35
**Melatonin AUC during light exposure**			
Intensity 1 (27 cd/m^2)	$F_{1,14}$ = 5.49, **p = .03**, ω_p^2=0.22		
Intensity 2 (62 cd/m^2)	$F_{1,13}$= 2.86, p = 0.11		
Intensity 3 (134 cd/m^2)	$F_{1,17}$= 4.69, **p < .05**, ω_p^2=0.16		
Intensity 4 (284 cd/m^2)	$F_{1,17}$= 4.70, **p < .05**, ω_p^2=0.16		
**Melatonin Onset**			
Intensity 1 (27 cd/m^2)	$F_{1,13.12}$ = 24.39, **p < .001**, ω_p^2=0.61		
Intensity 2 (62 cd/m^2)	$F_{1,14}$= 0.89, p = 0.36		
Intensity 3 (134 cd/m^2)	$F_{1,14.24}$= 5.77, **p = .03**, ω_p^2=0.23		
Intensity 4 (284 cd/m^2)	$F_{1,14.28}$= 9.31, **p = <.01**, ω_p^2=0.34		
**Subjective Sleepiness**			
Intensity 1 (27 cd/m^2)	$F_{1,254.03}$ = 21.69, **p < .001**, ω_p^2=0.07	$F_{7, 254.03}$ = 17.42, **p < .001**, ω_p^2=0.3	$F_{7,254.03}$ = 1.42, p = 0.20
Intensity 2 (62 cd/m^2)	$F_{1,255}$ = 14.68, **p < .001**, ω_p^2=0.05	$F_{7, 255}$ = 19.38, **p < .001**, ω_p^2=0.33	$F_{7,255}$= 0.54, p = 0.80
Intensity 3 (134 cd/m^2)	$F_{1,255}$ = 4.33, **p = 04**, ω_p^2=0.01	$F_{7, 255}$ = 29.38, **p < .001**, ω_p^2=0.43	$F_{7,255}$ = 0.39, p = 0.91
Intensity 4 (284 cd/m^2)	$F_{1,255}$ = 38.60, **p < .001**, ω_p^2=0.13	$F_{7, 255}$ = 18.27, **p < .001**, ω_p^2=0.31	$F_{7,255}$ = 0.11, p = 1.0

Supplementary Table 2: Results of the analysis of variance for sleep latency (log-transformed), melatonin concentration, melatonin AUC (Area Under the Curve), melatonin onset, subjective sleepiness results and effect sizes (ω_p^2). Effect sizes can be interpreted as follows: small effect: $\omega_p^2 \geq .01$, medium effect: $\omega_p^2 \geq .06$, large effect: $\omega_p^2 \geq .14$. A p-value < .05 was considered indicating statistical significance. In bold results with p < .05. Sleep latency: n = 18 per group, except LM intensity 1 n = 17; melatonin concentrations and AUC: intensity 1 n = 15, intensity 2 n = 14, intensity 3 n = 18, intensity 4 n = 18; melatonin onset: intensity 1 LM n = 15 and HM n = 17; intensity 2 LM n = 15 and HM n=15; intensity 3: LM n = 16 and HM n=16; intensity 4: LM n = 16 and HM n=15; subjective sleepiness: n = 18 per intensity group.

	Light Condition							
	**High Melanopic (HM)**				**Low Melanopic (LM)**			
**Sleep latency**	**N**	**Mean**	**95% CI- Lower Limit**	**95% CI- Upper Limit**	**N**	**Mean**	**95% CI- Lower Limit**	**95% CI- Upper Limit**
Intensity 1 (27 cd/m^2)	18	10.71	8.38	13.67	17	9.03	7.37	11.07
Intensity 2 (62 cd/m^2)	18	11.51	8.59	15.43	18	10.03	8.31	12.10
Intensity 3 (134 cd/m^2)	18	12.82	10.23	16.06	18	10.69	8.91	12.83
Intensity 4 (284 cd/m^2)	18	13.14	10.80	15.99	18	10.26	7.86	13.39
**Melatonin concentration during light exposure**								
Intensity 1 (27 cd/m^2)	15	7.88	6.35	9.41	15	6.74	5.59	7.89
Intensity 2 (62 cd/m^2)	14	6.71	2.10	11.32	14	5.00	2.13	7.86
Intensity 3 (134 cd/m^2)	18	6.42	4.49	8.35	18	5.32	3.54	7.10
Intensity 4 (284 cd/m^2)	18	5.28	3.44	7.13	18	4.11	2.37	5.84
**Melatonin concentration during the morning**								
Intensity 1 (27 cd/m^2)	15	4.92	3.01	6.83	15	4.28	2.77	5.79
Intensity 2 (62 cd/m^2)	14	7.14	4.73	9.54	14	7.87	5.86	9.88
Intensity 3 (134 cd/m^2)	18	7.02	4.28	9.76	18	7.53	5.00	10.06
Intensity 4 (284 cd/m^2)	18	7.25	5.47	9.03	18	6.09	4.14	8.04
**Melatonin AUC during light exposure**								
Intensity 1 (27 cd/m^2)	15	22.92	18.86	26.98	15	27.27	22.12	32.42
Intensity 2 (62 cd/m^2)	14	16.50	6.60	26.39	14	22.69	6.25	39.12
Intensity 3 (134 cd/m^2)	18	17.81	11.56	24.05	18	21.69	15.08	28.31
Intensity 4 (284 cd/m^2)	18	13.55	7.47	19.63	18	17.76	11.35	24.16
**Melatonin Onset**								
Intensity 1 (27 cd/m^2)	17	-2.52	-2.97	-2.08	15	-2.92	-3.36	-2.47
Intensity 2 (62 cd/m^2)	15	-2.05	-2.42	-1.68	15	-2.18	-2.49	-1.87
Intensity 3 (134 cd/m^2)	16	-2.17	-2.56	-1.78	16	-2.49	-2.85	-2.13
Intensity 4 (284 cd/m^2)	15	-1.86	-2.36	-1.35	16	-2.35	-2.79	-1.92
**Subjective Sleepiness**								
Intensity 1 (27 cd/m^2)	18	1.18	0.47	1.88	18	1.90	1.41	2.38
Intensity 2 (62 cd/m^2)	18	1.40	0.68	2.11	18	2.04	1.29	2.80
Intensity 3 (134 cd/m^2)	18	2.10	1.45	2.75	18	2.40	1.86	2.94
Intensity 4 (284 cd/m^2)	18	1.32	0.62	2.02	18	2.27	1.58	2.96

Supplementary Table 3: Overview of the numbers of participants, mean and 95% confidence intervals of the mean for reverse-transformed sleep latency, melatonin concentration, melatonin AUC (Area Under the Curve), melatonin onset, subjective sleepiness results.

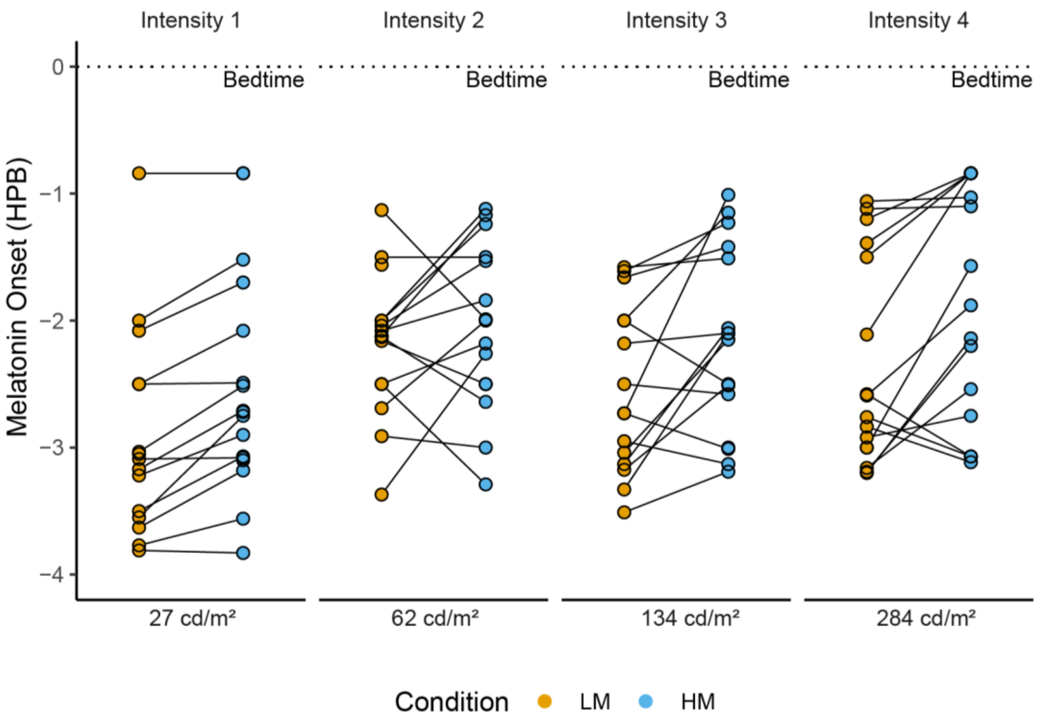

Supplementary Figure 1: Individual melatonin onsets relative to bedtime (HPB: Hours prior bedtime) during LM (orange points) and HM (blue points). Intensity 1: LM n = 15, HM n = 17; intensity 2: LM n = 15, HM n=15, intensity 3: LM n = 16, HM n=16; intensity 4: LM n = 16, HM n=15.

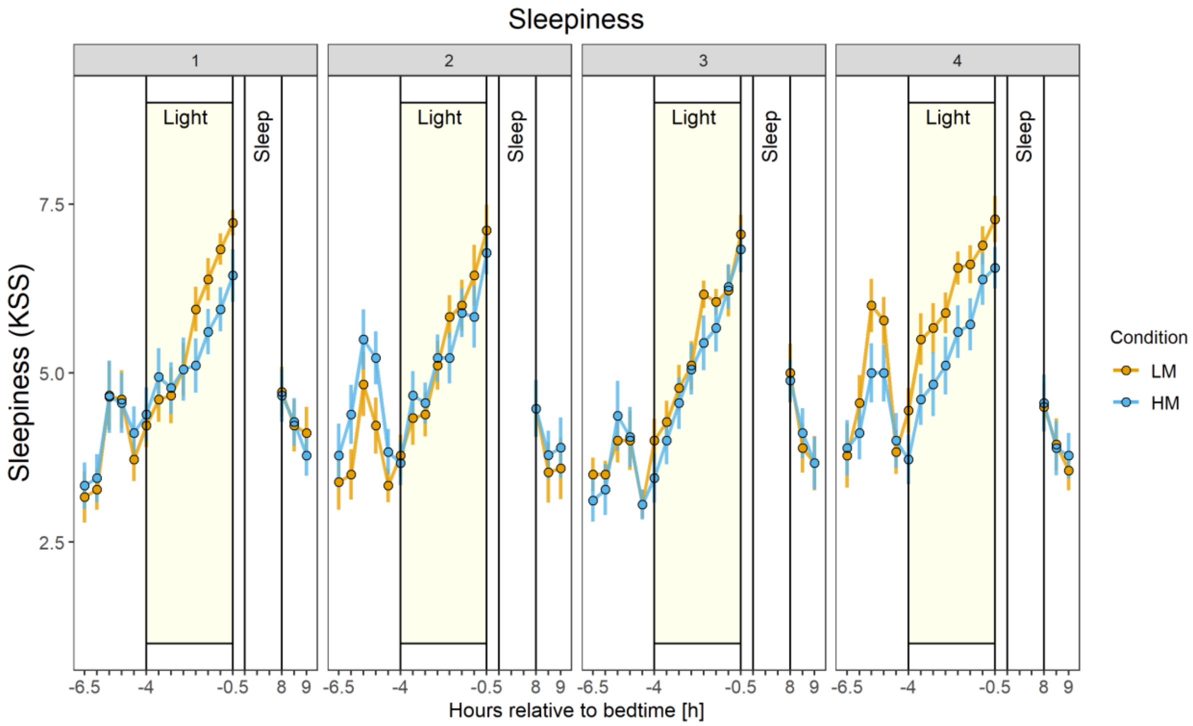

Supplementary Figure 2: Results subjective sleepiness ratings. Time course of subjective sleepiness ratings (KSS) (n = 18 per intensity group.) during the low melanopic (LM: orange points and lines) and high melanopic (HM: blue points and lines) light conditions plotted against the hours relative to bedtime [h]. Depicted are means ±1SEM.

**Before light exposure**	**Light condition (LM vs HM)**	**Time**	**Light condition * Time**
Intensity 1 (27 cd/m^2)	$F_{1,153}$ = 0.38, p = 0.54	**$F_{4, 153}$ = 7.53, p < .001**	$F_{4,153}$ = 0.13, p = 0.97
Intensity 2 (62 cd/m^2)	**$F_{1,153}$ = 9.18, p < .01**	**$F_{4, 153}$ = 7.86, p < .001**	$F_{4,153}$ = 0.25, p = 0.90
Intensity 3 (134 cd/m^2)	$F_{1,152.09}$ = 0.05, p = 0.83	**$F_{4, 152.09}$ = 7.86, p < .001**	$F_{4,152.09}$ = 0.43, p = 0.80
Intensity 4 (284 cd/m^2)	$F_{1,151.91}$ = 2.92, p = 0.09	**$F_{4, 151.91}$ = 11.21, p < .001**	$F_{4,151.91}$ = 1.04, p = 0.39
**Last session before light exposure**			
Intensity 1 (27 cd/m^2)	$F_{1,17}$ = 0.66, p = 0.43		
Intensity 2 (62 cd/m^2)	$F_{1,17}$ = 1.58, p = 0.42		
Intensity 3 (134 cd/m^2)	$F_{1,17}$ = 0, p = 1		
Intensity 4 (284 cd/m^2)	$F_{1,17}$ = 0.16, p = 0.70		
**Morning**			
Intensity 1 (27 cd/m^2)	$F_{1,85}$ = 0.46, p = 0.50	**$F_{2, 85}$ = 7.92, p = .002**	$F_{2,85}$ = 0.50, p = 0.61
Intensity 2 (62 cd/m^2)	$F_{1,85}$ = 0.51, p = 0.48	**$F_{2, 85}$ = 6.08, p = .003**	$F_{2,85}$ = 0.07, p = 0.95
Intensity 3 (134 cd/m^2)	$F_{1,85}$ = 0.05, p = 0.83	**$F_{2, 85}$ = 20.12, p < .001**	$F_{2,85}$ = 0.33, p = 0.72
Intensity 4 (284 cd/m^2)	$F_{1,85}$ = 0.11, p = 0.74	**$F_{2, 85}$ = 5.44, p = .006**	$F_{4,85}$ = 0.16, p = 0.87

Supplementary Table 4: Results of the analysis of variance for subjective sleepiness ratings before light exposure and in the morning without baseline correction. N = 18 per intensity group.

Supplementary Note 1: Cone Intrusion

Light conditions might not have been metameric for each participant, as the spectra were matched to the 10° Standard Observer and not individually adjusted. Using the same light conditions allowed us to keep the nominal luminance, colour and melanopsin contrast constant across the participants in the four light intensity groups.

We addressed the question of possible intrusions of cone signals into our stimulus following the splatter methodology developed by Spitschan et al. (2015, 2017).[4,5] As expected, the median contrast seen across the sample of observers is centred at or near 0 across all cone classes (see Supplementary Figure S3). However, the range of contrasts depends on the cone class, with the largest variability seen for the S cones, where some biologically plausible observers may have been exposed to up to |50%| contrast. Importantly, however, in the population of samples we find that the distribution is nonetheless centered around 0, rendering it unlikely that our melatonin-suppressive result can be explained by cone intrusion.

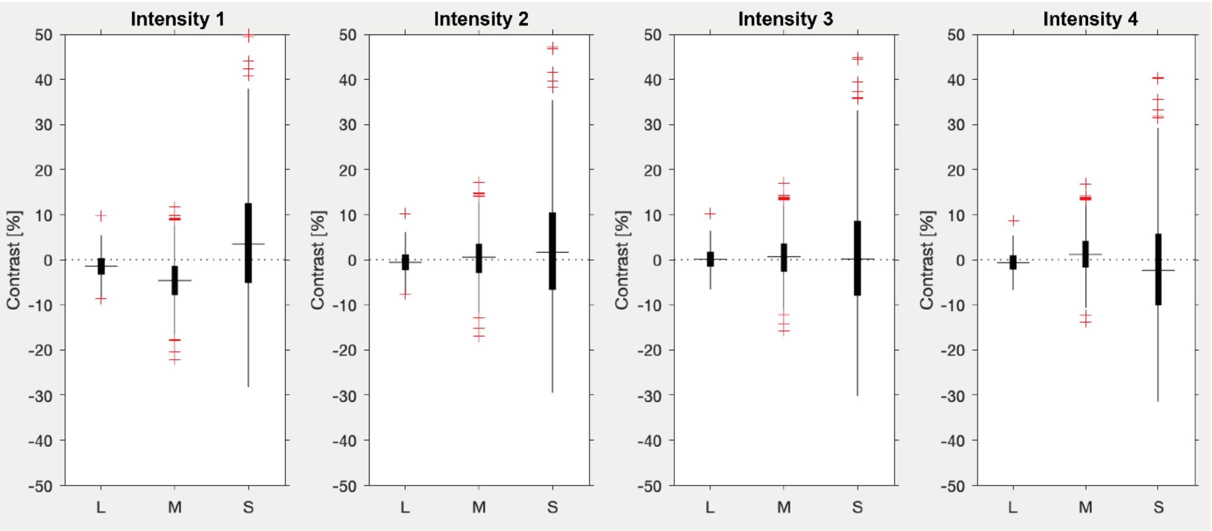

Supplementary Figure 3: Boxplot cone contrast [%] for generated observers following the splatter methodology. With Asano's individual colorimetric observer model[6], which includes individual-difference parameters for the cone spectral sensitivities, we generated 1000

biologically plausible observers based on the assumed distributions of these individual-difference parameters. We then calculated for each of these observers the contrast 'seen' by each observer's set of cones.[4,5] In the boxplot, whiskers extend to a maximum of 1.5 × interquartile range beyond the box, with a line at the median.

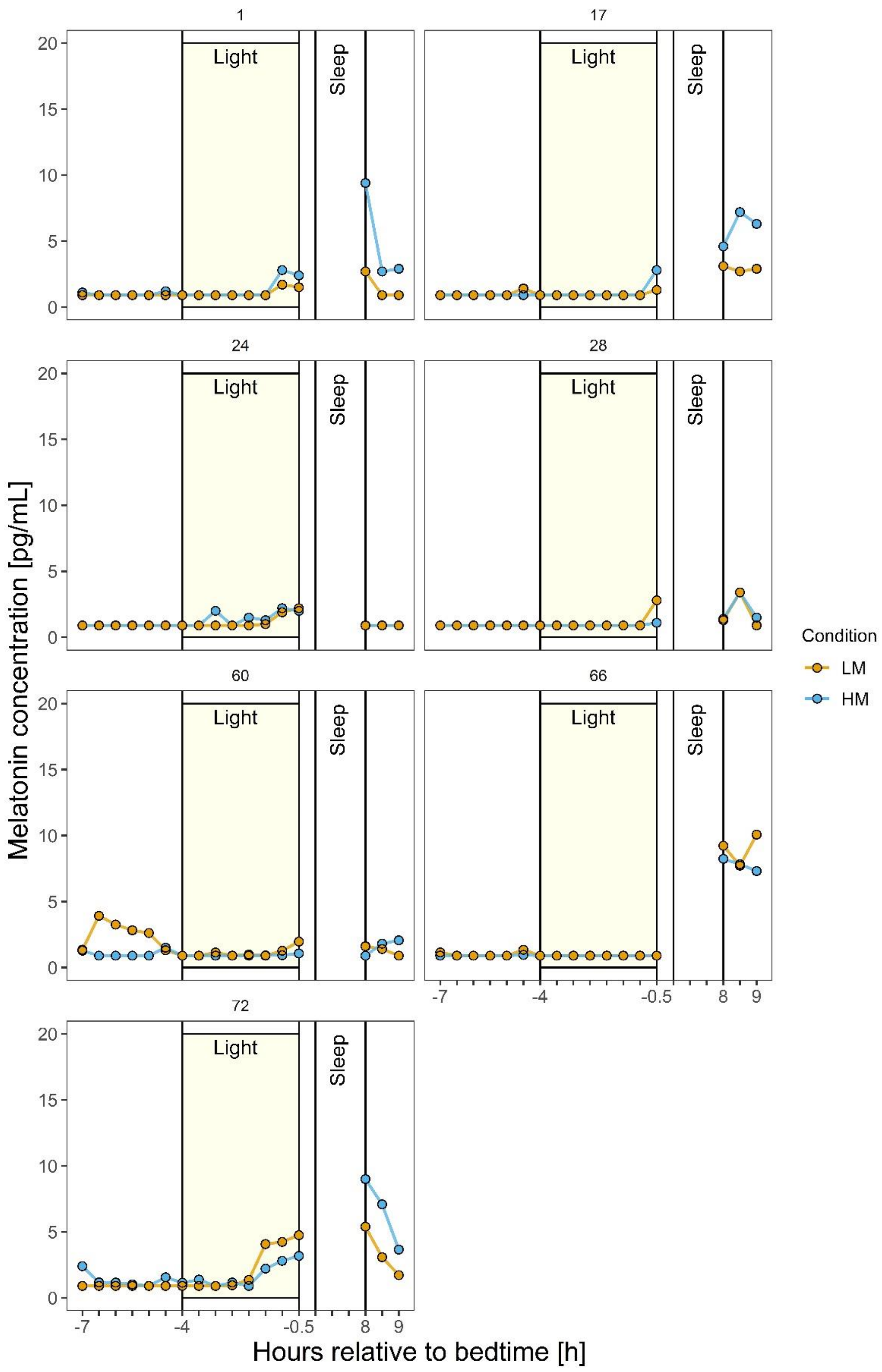

Supplementary Figure 4: Individual time courses of salivary melatonin concentrations in pg/mL of participants excluded for melatonin concentration and melatonin AUC analysis

during low melanopic (LM: orange points) and high melanopic (HM: blue points) conditions, plotted against hours relative to bedtime [h].

Light history and Season

Intensity	Condition	N	Mean time spend outdoors [min]	sd
1	LM	17	61.24	70.24
1	HM	17	70.00	67.41
2	LM	18	80.00	51.73
2	HM	17	71.18	54.57
3	LM	18	50.56	39.59
3	HM	18	57.22	57.27
4	LM	18	50.56	48.50
4	HM	18	37.50	21.85

Supplementary Table 5: Time spent outdoors during experimental days. Data are expressed as mean and sd.

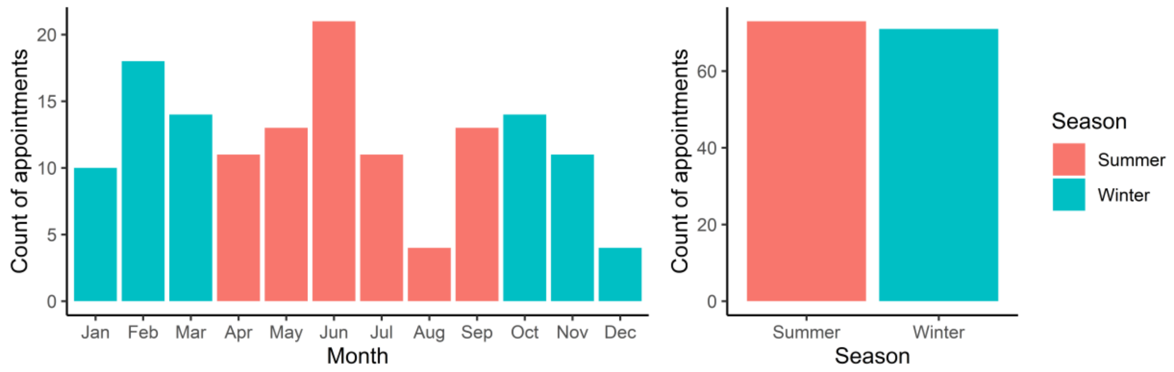

Supplementary Figure 5: Monthly and seasonal distribution of study appointments (n=144). Bars for the number of appointments in the winter months are shown in turquoise and in the sommer months in red.

Rod intrusion

	LM1	HM1	LM2	HM2	LM3	HM3	LM4	HM4
Luminance [cd/m²]	27	27	62	62	134	134	284	284
Pupilsize [mm]	4	4	3.6	3.6	3.2	3.2	3	3
Scotopic Retinal Illuminance Trolands [Ts]	187	509	360	890	663	1511	1331	2767
Log10 scotopic Retinal Illuminance [log Ts]	2.27	2.71	2.56	2.95	2.82	3.18	3.12	3.44

Supplementary Table 6: Scotopic retinal illuminance for evaluation of rod saturation. For the simulation of pupil size, the model for light-adapted pupil size of Watson and Yellott was used.[1] The measured luminance and a solid angle of 30 degrees were used for the calculation. According to Aguilar and Stiles (1954), rod saturation occurs between 2000 and 5000 scotopic trolands [Ts] (corresponds to 3.3 and 3.7 log Ts)[2] and according Andelson (1982) rod saturation occurs between 2.5 and 3.0 log Ts (corresponds to 316 and 1000 scotopic Ts)[3]. Therefore, we assume that the light effects in intensity groups 2 to 4 did not occur because of the rod intrusion.

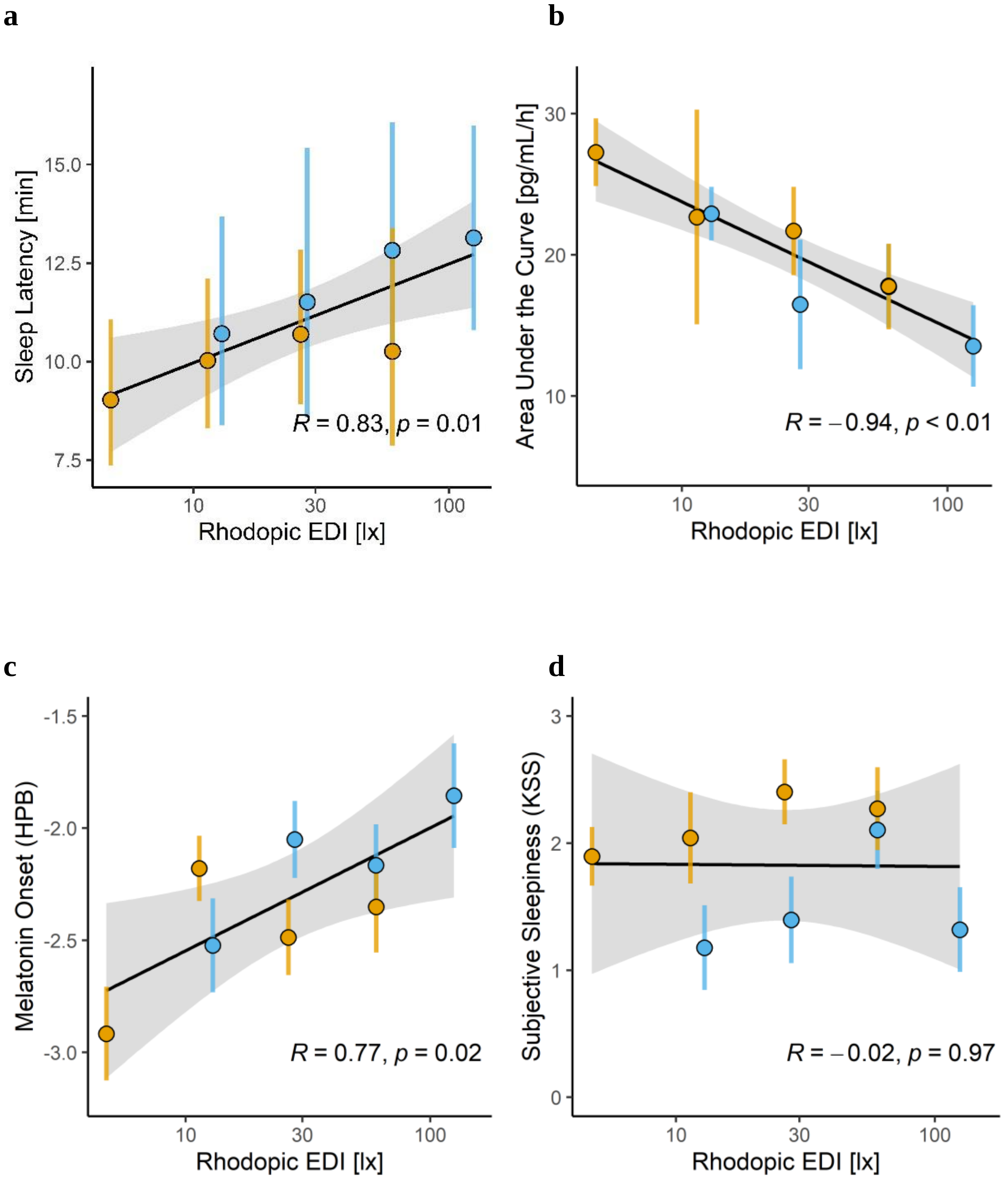

Supplementary Figure 6: Dose response relationships with log10-transformed rhodopic EDI [lx]. Averages per light condition over all light intensity groups entered calculation of the regression models. Dose-dependencies were calculated for **a** Sleep latency [min], depicted are retransformed means and 95% CIs (n = 18 per group, except LM intensity 1 n = 17) and the regression line (Mean values of all light conditions, n=8), **b** Melatonin Area under the Curve (AUC) [pg/mL/h], depicted are means±1SEM groups (Intensity 1: n = 15, intensity 2: n = 14, intensity 3: n = 18, intensity 4: n = 18) and the regression line (Mean values of all light

conditions, n=8), **c** Melatonin Onset in hours prior individual bedtime (HPB), depicted are means±1SEM (Intensity 1: LM n = 15, HM n = 17; intensity 2: LM n = 15, HM n=15; intensity 3: LM n = 16, HM n=16; intensity 4: LM n = 16, HM n=15) and the regression line (Mean values of all light conditions, n=8), **d** Baseline-corrected subjective sleepiness (KSS), depicted are means±1SEM (n = 18 per intensity group) and the regression line (Mean values of all light conditions, n=8). Orange points show means of the low melanopic and orange lines 95% CIs or SEM. Blue points depict means of the high melanopic and blue lines 95% CIs or SEM. The grey bands represent the 95% confidence interval limits.

Light Conditions

Condition	LM 1	HM 1	LM 2	HM 2	LM 3	HM 3	LM 4	HM 4
**Luminance [cd/m^2]**	27.43	27.41	62.93	61.39	135.03	133	283.74	284.5
**Illuminance [lx]**	8.61	7.18	19.45	15.94	41.96	34.36	88.63	73.66
**CIE 1964 $x_{10}y_{10}$ chromaticity (x_{10})**	0.34	0.33	0.33	0.34	0.34	0.34	0.34	0.34
**CIE 1964 $x_{10}y_{10}$ chromaticity (y_{10})**	0.33	0.33	0.34	0.33	0.34	0.33	0.34	0.33
**S-cone-opic irradiance (mW · m^{-2})**	6.40	6.22	13.87	13.72	29.58	29.71	60.21	62.00
**M-cone-opic irradiance (mW · m^{-2})**	11.19	11.74	25.52	25.43	55.08	54.79	116.58	115.12
**L-cone-opic irradiance (mW · m^{-2})**	13.32	13.50	29.95	30.09	64.90	64.78	138.10	138.86
**Rhodopic irradiance (mW · m^{-2})**	6.87	18.70	16.47	40.39	38.09	86.81	87.00	180.89
**Melanopic irradiance (mW · m^{-2})**	4.91	20.11	11.70	43.51	27.46	93.27	64.17	193.62
**S-cone-opic EDI [lx]**	7.83	7.61	16.97	16.79	36.19	36.36	73.67	75.86
**M-cone-opic EDI [lx]**	7.69	8.07	17.53	17.47	37.83	37.63	80.08	79.08
**L-cone-opic EDI [lx]**	8.18	8.29	18.39	18.47	39.84	39.77	84.78	85.25
**Rhodopic EDI [lx]**	4.74	12.90	11.36	27.86	26.28	59.88	60.01	124.78
**mEDI [lx]**	3.70	15.17	8.82	32.81	20.70	70.33	48.39	146.00

Supplementary Table 7: Overview light measures of the experimental light conditions.

Melatonin analyses with all participant data

**Melatonin concentration during light exposure**	*Condition*	*Sample*	*Condition*Sample*
Intensity 1 (27 cd/m^2)	$F_{1,255}$ = 5.47, **p =.02**, ω_p^2=0.02	$F_{7,255}$ = 58.67, **p < .001**, ω_p^2=0.61	$F_{7,255}$ = 0.41, p = 0.90
Intensity 2 (62 cd/m^2)	$F_{1,255}$ = 4.95, **p = .03**, ω_p^2=0.02	$F_{7,255}$ = 21.24, **p < .001**, ω_p^2=0.35	$F_{7,255}$ = 0.27, p = 0.97
Intensity 3 (134 cd/m^2)	$F_{1,254.01}$ = 8.33, **p < .01**, ω_p^2=0.03	$F_{7,254.01}$ = 61.82, **p < .001**, ω_p^2=0.62	$F_{7,254.01}$ = 1.12, p = 0.35
Intensity 4 (284 cd/m^2)	$F_{1,254.02}$ = 8.95, **p < .01**, ω_p^2=0.03	$F_{7,254.02}$ = 38.58, **p < .001**, ω_p^2=0.5	$F_{7,254.02}$ = 1.10, p = 0.36
**Melatonin concentration in the morning**			
Intensity 1 (27 cd/m^2)	$F_{1,85}$ = 11.34, **p = .001**, ω_p^2=0.11	$F_{2,85}$ = 16.03, **p < .001**, ω_p^2=0.25	$F_{2,85}$ = 1.34, p = 0.27
Intensity 2 (62 cd/m^2)	$F_{1,85}$ = 0.64, p = 0.43	$F_{2,85}$ = 17.69, **p < .001**	$F_{2,85}$ = 0.10, p = 0.91
Intensity 3 (134 cd/m^2)	$F_{1,85}$ = 1.17, p = 0.28	$F_{2,85}$ = 15.29, **p < .001**	$F_{2,85}$ = 0.12, p = 0.89
Intensity 4 (284 cd/m^2)	$F_{1,85}$ = 9.04, **p < .01**, ω_p^2=0.08	$F_{2,85}$ = 23.42, **p < .001**, ω_p^2=0.34	$F_{2,85}$ = 1.06, p = 0.35
**Melatonin AUC during light exposure**			
Intensity 1 (27 cd/m^2)	$F_{1,17}$ = 5.06, **p = .04,** ω_p^2=0.18		
Intensity 2 (62 cd/m^2)	$F_{1,17}$= 2.87, p = 0.11		
Intensity 3 (134 cd/m^2)	$F_{1,17}$= 4.69, **p < .05,** ω_p^2=0.16		
Intensity 4 (284 cd/m^2)	$F_{1,17}$= 4.70, **p < .05,** ω_p^2=0.16		

Supplementary Table 8: Results of the analysis of variance for melatonin concentration and melatonin AUC (Area Under the Curve) of the full data set (n=18 per group). In bold results with p <.05.

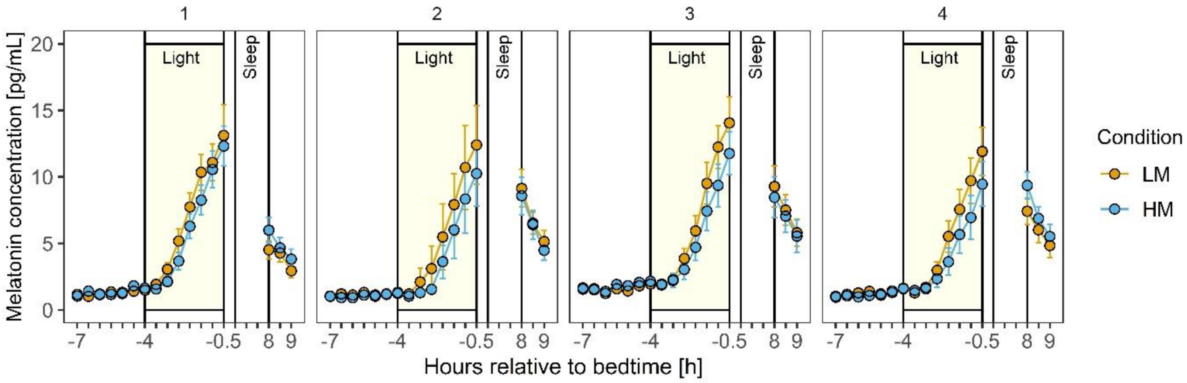

Supplementary Figure 7: Results for melatonin during light exposure. a Time course of salivary melatonin concentrations in pg/mL during the low melanopic (LM: orange) and high melanopic (HM: blue) light conditions plotted against the hours relative to bedtime [h]. Depicted are means ±1SEM of the four light intensity groups (Intensity 1: n=18, Intensity 2: n=18, Intensity 3: n=18, Intensity 4: n=18).

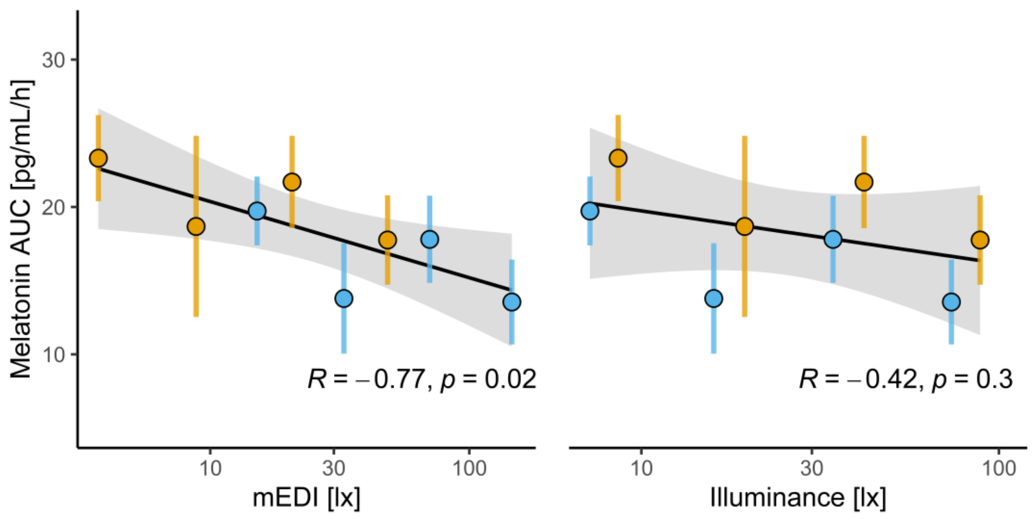

Supplementary Figure 8: Dose response relationship of melatonin Area under the Curve (AUC) [pg/mL/h] with log10-transformed mEDI [lx] and photopic illuminance [lx]. Depicted are means±1SEM (Intensity 1: n=18, Intensity 2: n=18, Intensity 3: n=18, Intensity 4: n=18) and the regression line, which was calculated with the averages per light condition over all light intensity groups (n=8). The grey bands represent the 95% confidence interval limits.

Supplementary References

1. Watson, A. B. & Yellott, J. I. A unified formula for light-adapted pupil size. *Journal of Vision* **12**, 12 (2012).
2. Aguilar, M. & Stiles, W. S. Saturation of the Rod Mechanism of the Retina at High Levels of Stimulation. *Optica Acta: International Journal of Optics* **1**, 59–65 (1954).
3. Adelson, E. H. Saturation and adaptation in the rod system. *Vision Research* **22**, 1299–1312 (1982).
4. Spitschan, M. *et al.* The human visual cortex response to melanopsin-directed stimulation is accompanied by a distinct perceptual experience. *Proc Natl Acad Sci USA* **114**, 12291–12296 (2017).
5. Spitschan, M., Aguirre, G. K. & Brainard, D. H. Selective Stimulation of Penumbral Cones Reveals Perception in the Shadow of Retinal Blood Vessels. *PLoS ONE* **10**, e0124328 (2015).
6. Asano, Y., Fairchild, M. D. & Blondé, L. Individual Colorimetric Observer Model. *PLoS ONE* **11**, e0145671 (2016).
