# Supplementary material for: Melanopic irradiance defines the impact of evening display light on sleep latency, melatonin and alertness
Source: Commun Biol. 2023 Mar 1;6:228. doi: 10.1038/s42003-023-04598-4 (PMC9974389; doi:10.1038/s42003-023-04598-4)
Supplement: Supplementary file 4 — Reporting Summary [file 42003_2023_4598_MOESM4_ESM.pdf]

nature portfolio

Corresponding author(s): Christian Cajochen

Last updated by author(s): Jan 28, 2023

Reporting Summary

Nature Portfolio wishes to improve the reproducibility of the work that we publish. This form provides structure for consistency and transparency in reporting. For further information on Nature Portfolio policies, see our Editorial Policies and the Editorial Policy Checklist.

Statistics

For all statistical analyses, confirm that the following items are present in the figure legend, table legend, main text, or Methods section.

n/a	Confirmed	
☐	☒	The exact sample size (*n*) for each experimental group/condition, given as a discrete number and unit of measurement
☐	☒	A statement on whether measurements were taken from distinct samples or whether the same sample was measured repeatedly
☐	☒	The statistical test(s) used AND whether they are one- or two-sided<br>*Only common tests should be described solely by name; describe more complex techniques in the Methods section.*
☐	☒	A description of all covariates tested
☒	☐	A description of any assumptions or corrections, such as tests of normality and adjustment for multiple comparisons
☐	☒	A full description of the statistical parameters including central tendency (e.g. means) or other basic estimates (e.g. regression coefficient) AND variation (e.g. standard deviation) or associated estimates of uncertainty (e.g. confidence intervals)
☐	☒	For null hypothesis testing, the test statistic (e.g. *F*, *t*, *r*) with confidence intervals, effect sizes, degrees of freedom and *P* value noted<br>*Give P values as exact values whenever suitable.*
☒	☐	For Bayesian analysis, information on the choice of priors and Markov chain Monte Carlo settings
☐	☒	For hierarchical and complex designs, identification of the appropriate level for tests and full reporting of outcomes
☐	☒	Estimates of effect sizes (e.g. Cohen's *d*, Pearson's *r*), indicating how they were calculated

Our web collection on statistics for biologists contains articles on many of the points above.

Software and code

Policy information about availability of computer code

Data collection	Software testbattery: PsychoPy version 3.1.5<br>Program for running light conditions: DMXControl
Data analysis	All statistical analysis and data visualizations were conducted in R (Version 4.1.1, R Core Team, 2021).). Linear mixed models were calculated using the packages lme4 and lmerTest. As effect size measure partial omega squared (ωp2) was calculated by the effectsize package.

For manuscripts utilizing custom algorithms or software that are central to the research but not yet described in published literature, software must be made available to editors and reviewers. We strongly encourage code deposition in a community repository (e.g. GitHub). See the Nature Portfolio guidelines for submitting code & software for further information.

Data

Policy information about availability of data

All manuscripts must include a data availability statement. This statement should provide the following information, where applicable:

- Accession codes, unique identifiers, or web links for publicly available datasets
- A description of any restrictions on data availability
- For clinical datasets or third party data, please ensure that the statement adheres to our policy

We added a supplemental .xlsx file that contains spectral distributions of irradiance and radiance of all high and low melanopic light conditions and source data underlying the graphs presented in the main figures.

Human research participants

Policy information about studies involving human research participants and Sex and Gender in Research.

Reporting on sex and gender	Male participants.
Population characteristics	Healthy males between 19 and 35 years were included in the study. Exclusion criteria comprised body mass index (BMI) < 19 or > 26, medication, drug and nicotine consumption, shift work < 3 months and transmeridian travel (> 2 time zones) < 1 month prior study start, mother tongue other than German. People with an extreme Chronotypes (MEQ score: ≤ 30 and ≥ 70), poor sleep quality (PSQI score > 5), poor sleep efficiency (SE < 70 %) and sleep disorders like sleep apnea (apnea index > 10), as well as periodic limb movements (PLMS > 15) were excluded from the study. Furthermore, participants with a monocular visual acuity < 0.5, with color vision deficiencies (Ishihara < 17 of 21 plates, 100-hue error score > 40) and with reduced stereoscopic vision (Lang II < 200 arc seconds) were not included in the study.
Recruitment	Advertisement on the university homepage.
Ethics oversight	The experimental protocol, screening questionnaires and consent form were approved by the Ethics Committee northwest/ central Switzerland (2019-00571)

Note that full information on the approval of the study protocol must also be provided in the manuscript.

Field-specific reporting

Please select the one below that is the best fit for your research. If you are not sure, read the appropriate sections before making your selection.

☒ Life sciences ☐ Behavioural & social sciences ☐ Ecological, evolutionary & environmental sciences

For a reference copy of the document with all sections, see nature.com/documents/nr-reporting-summary-flat.pdf

Life sciences study design

All studies must disclose on these points even when the disclosure is negative.

Sample size	total = 72 (n=18 per light intensity group)
Data exclusions	Seven participants in the light intensity groups 1-3 were excluded from further melatonin concentration analyses because the maximal evening melatonin levels were below 5 pg/mL. Data analyses of the full dataset can be found in the Supplemental Information. In nineteen evening melatonin profiles, we could not detect any melatonin onset during light exposure. Due to technical issues we lost one night of EEG recording.
Replication	No replication.
Randomization	72 participants were assigned to one of four light intensity groups (n=18 per group) and each participant was exposed to a low and high melanopic radiance setting (LM and HM respectively) in balanced randomised order.
Blinding	Data collection was not blinded because the study investigators had to adjust the light conditions manually. The manual sleep scorings were blinded, and the analyses of the melatonin samples were done by a third party blinded by the treatment.

Reporting for specific materials, systems and methods

We require information from authors about some types of materials, experimental systems and methods used in many studies. Here, indicate whether each material, system or method listed is relevant to your study. If you are not sure if a list item applies to your research, read the appropriate section before selecting a response.

Materials & experimental systems

n/a	Involved in the study	
☒	☐	Antibodies
☒	☐	Eukaryotic cell lines
☒	☐	Palaeontology and archaeology
☒	☐	Animals and other organisms
☒	☐	Clinical data
☒	☐	Dual use research of concern

Methods

n/a	Involved in the study	
☒	☐	ChIP-seq
☒	☐	Flow cytometry
☒	☐	MRI-based neuroimaging
